# *Strongyloides* Colitis as a Harmful Mimicker of Inflammatory Bowel Disease

**DOI:** 10.1155/2017/2560719

**Published:** 2017-05-07

**Authors:** Julio Poveda, Farah El-Sharkawy, Leopoldo R. Arosemena, Monica T. Garcia-Buitrago, Claudia P. Rojas

**Affiliations:** ^1^Department of Pathology, University of Miami Health System/Jackson Memorial Hospital, 1611 NW 12th Ave, Holtz Center 2142D, Miami, FL 33136, USA; ^2^Department of Gastroenterology, University of Miami Health System/Jackson Memorial Hospital, 1475 NW 12th Ave, Miami, FL 33136, USA

## Abstract

Autoinfection caused by* Strongyloides stercoralis *frequently becomes a life-long disease unless it is effectively treated. There is overlapping histomorphology between* Strongyloides *colitis and inflammatory bowel disease; a low index of suspicion can lead to misdiagnosis and fatal consequences. We present a case of* Strongyloides *colitis mimicking the clinical and pathologic features of inflammatory bowel disease. A 64-year-old female presented to the emergency department with a four-day history of abdominal pain, diarrhea, and hematochezia. Colonoscopy revealed diffuse inflammation suggestive of inflammatory bowel disease, which led to initiation of 5-aminosalicylic acid and intravenous methylprednisolone. Biopsies of the colon revealed increased lymphoplasmacytic infiltrate of the lamina propria with eosinophilic microabscesses and presence of larvae, consistent with* Strongyloides stercoralis*. Immunosuppressive medication was halted. The patient ultimately died a few days later. This case emphasizes the importance of identifying the overlapping clinical and pathologic features of* Strongyloides *colitis and inflammatory bowel disease. A high index of suspicion and recognition of particular histological findings, including eosinophilic microabscesses, aid in the correct diagnosis. Definitive diagnosis is crucial as each disease carries distinct therapeutic implications and outcome.

## 1. Introduction

Strongyloidiasis is an infectious disorder caused by the nematode* Strongyloides stercoralis*, which is endemic in subtropical and tropical regions with poor sanitary conditions. In the United States, this infection is prevalent in several rural areas of the southeast and Appalachian region. The condition may be asymptomatic in immunocompetent patients, or it may manifest with occasional stomachache, intermittent diarrhea and constipation, bloating, nausea, and loss of appetite. Severe life-threatening complications of hyperinfection syndrome and disseminated strongyloidiasis may develop in patients with HTLV-1 confection or in patients receiving immunosuppressive therapy, such as corticosteroids [1–3].

## 2. Case Report

A 64-year-old Hispanic immigrant woman presented to the emergency department complaining of abdominal pain, hematochezia, and episodes of diarrhea that began four days prior to admission. She had a significant past medical history of diabetes mellitus, hypertension, rheumatoid arthritis, and diverticulosis. The patient had a general healthy appearance and was in no acute distress. Initial lab results reported a high leucocyte count (16,000/mm^3^), eosinophilia (9.3%), anemia with hemoglobin of 9.9 g/dL, and hematocrit of 31.4%.

**Figure 1:**
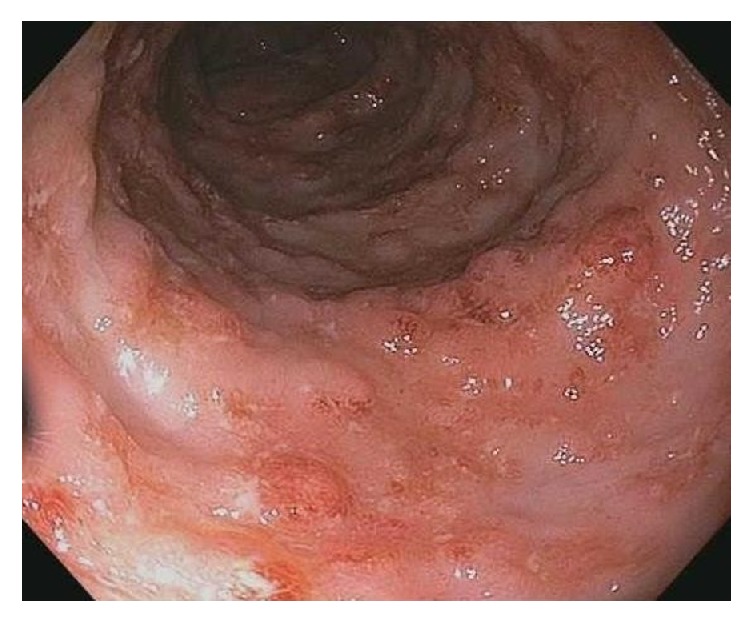
Colonoscopy showing portion of ascending colon with congested mucosa and aphthous and confluent ulcerations.

**Figure 2:**
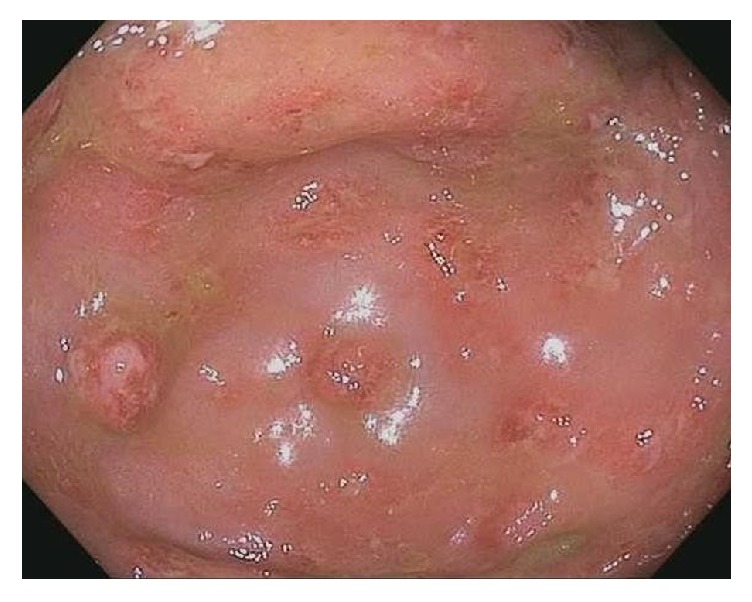
Colonoscopy showing portion of cecum with multiple aphthous ulcers.

The colonoscopy revealed inflammation characterized by congestion, edema, erythema, friability, and aphthous and confluent ulcerations throughout the entire colon (Figures 1 and 2). As these findings were suggestive of ulcerative colitis, a combination of oral 5-aminosalicylic acid and intravenous methylprednisolone was started. After two days of therapy, the patient suddenly became lethargic, tachycardic, and hypotensive. She was intubated for airway protection and was transferred to the intensive care unit.

**Figure 3:**
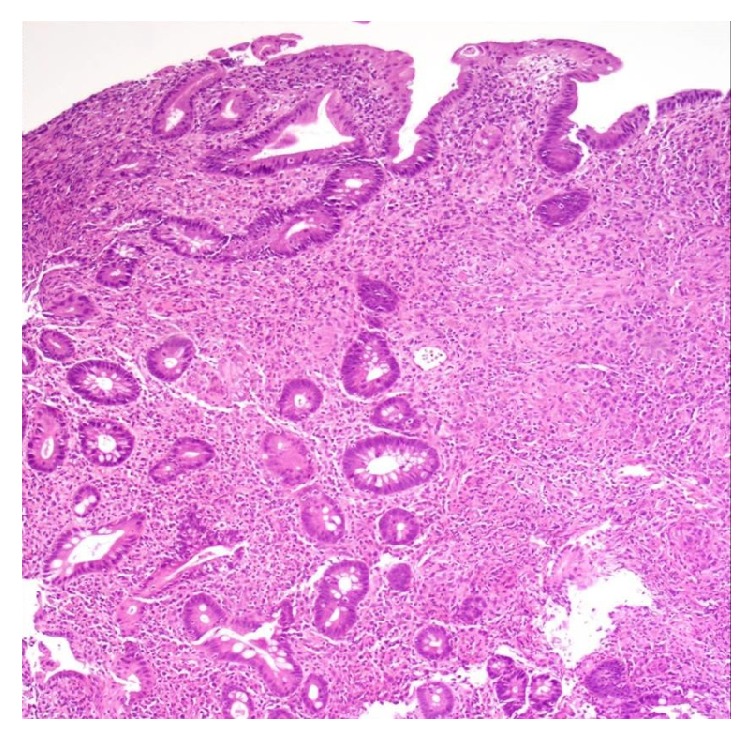
Colonic mucosa showing marked architectural distortion and crypt branching, increased lymphoplasmacytic and eosinophilic infiltrate in the lamina propria, and cryptitis. H&E 10x.

**Figure 4:**
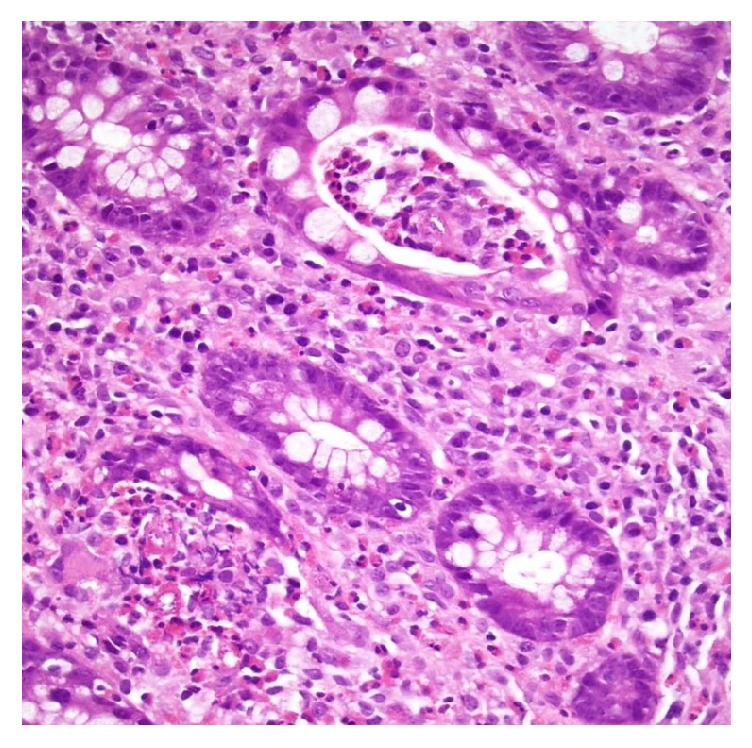
Eosinophilic microabscesses within glands and lymphoplasmacytic infiltrate in the lamina propria. H&E 20x.

**Figure 5:**
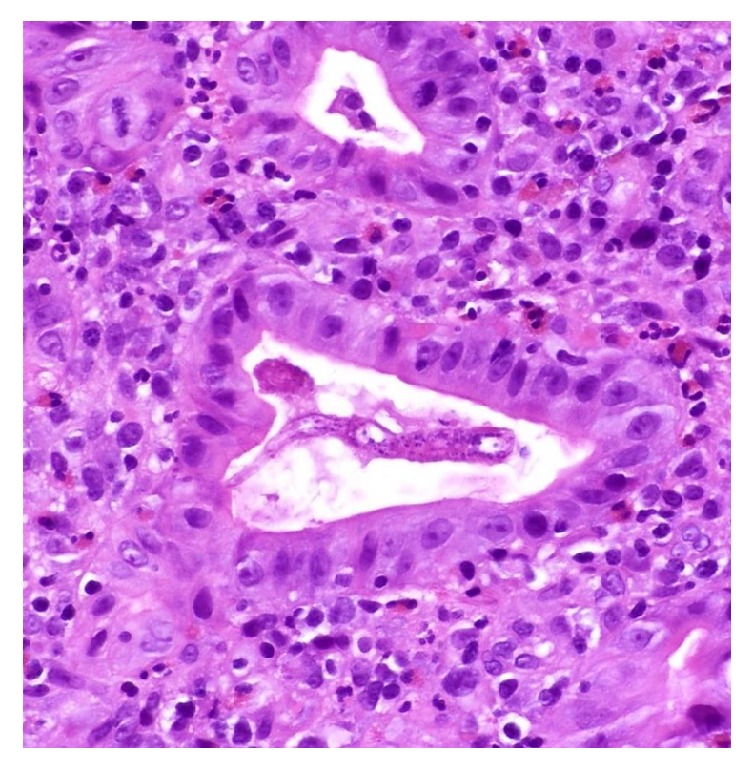
*Strongyloides *larva in the lumen of a crypt. H&E 40x.

Blood cultures returned positive for Gram-negative rods. Biopsies from colonoscopy revealed lymphoplasmacytic infiltrate of the lamina propria with mild architectural distortion, eosinophilic microabscesses, crypt abscesses, and presence of larvae, representative of* Strongyloides *infection (Figures 3–5). A diagnosis of strongyloidiasis and sepsis was made. Treatment with vancomycin, meropenem, metronidazole, fluconazole, valacyclovir, and ivermectin was initiated, and immunosuppressive therapy was halted. The patient was maintained on vasopressors and aggressive fluid hydration due to hemodynamic instability. The patient subsequently died due to the systemic complications of strongyloidiasis.

## 3. Discussion

*Strongyloides stercoralis *is unique in its ability to exist and replicate within a host for decades while remaining asymptomatic, or producing minimal nonspecific symptoms, until it transitions to a potentially fatal disseminated infection. Risk factors to these complications are immunosuppression, corticosteroid therapy, transplantation, malnutrition, alcoholism, and HTLV-1 coinfection [1–6]. HTLV-1 coinfection is a particularly strong risk factor for severe forms of strongyloidiasis due to an impaired Th2 immune response [1, 5, 6].

Infection occurs via penetration of larvae into the skin or mucous membranes from soil or feces. Once in the tissue, larvae enter the circulation and migrate into the alveolar spaces. The larvae ascend through the respiratory tract and are swallowed by the host, which leads them to the gastrointestinal tract. In the small bowel, larvae mature into adult females, which produce eggs through parthenogenesis. The excretion of larvae constitutes the mainstay of diagnosis via laboratory examination of stool [1, 2].

A unique characteristic of* S. stercoralis *is its ability to maintain an autoinfective cycle. Larvae reenter the circulation by invading the intestinal mucosa, or they may penetrate the perianal skin. Autoinfection occurs in hosts with an impaired cell-mediated immune response. The immunocompromised state allows for the development of the most severe forms of strongyloidiasis: hyperinfection syndrome and disseminated strongyloidiasis [1–3]. The mortality associated with these conditions can be as high as 87% [7].

Hyperinfection syndrome is a severe complication of longstanding infection in which there is an excessive increase in the worm load within the host. The most common risk factor is corticosteroid therapy [3, 7]. Disseminated strongyloidiasis is characterized by abundant widespread larvae to extraintestinal sites, such as the lungs, heart, kidneys, central nervous system, and endocrine organs [4]. In the course of severe disseminated disease or hyperinfection, a patient may test positive for enteric or Gram-negative bacteremia due to the translocation of gut bacteria through an ulcerated mucosa [2, 8]. Our patient suffered from sepsis as a complication of hyperinfection syndrome and disseminated strongyloidiasis following immunosuppressive therapy.

The macroscopic findings of strongyloidiasis on endoscopy are frequently confused with those of ulcerative colitis or Crohn's disease. Upper endoscopy usually reveals hyperemic edematous duodenal mucosa, erythema, friability, and white villi in the duodenum. Colonoscopy may show mucosal edema, erosions, submucosal hemorrhage, and ulcerations, which alternate with portions of normal mucosa [8–10]. The distinction between strongyloidiasis and inflammatory bowel disease (IBD) is made microscopically; however, there are significant overlapping features between both entities. Histological examination in strongyloidiasis reveals edema and infiltration of the lamina propria by lymphocytes, plasma cells and eosinophils, blunted villi, cryptitis, and crypt hyperplasia, which are also common findings in IBD [8, 9, 11]. In contrast to ulcerative colitis, inflammation caused by* Strongyloides *almost always extends into the submucosa and can be transmural; it often skips some areas in the involved segments (skip lesions), rarely involves the rectum, and shows milder crypt architecture distortion. Attenuation of the disease in the distal colon and rectum is common in strongyloidiasis, while ulcerative colitis characteristically affects the distal colon and rectum [11]. The skip lesions and eosinophilic granulomas found in strongyloidiasis are also found in Crohn's disease. However, the transmural granulomatous inflammatory process in strongyloidiasis is caused by the presence of larvae, a pathognomonic finding [11, 12]. The granulomas often have abundant histiocytes or may have mature giant cells containing the remains of larvae [12]. Another distinctive feature is the formation of eosinophilic microabscesses in the lamina propria and submucosa [11].

Treatment for* S*.* stercoralis *colitis is drastically different from that for IBD. Antihelminthic therapy with ivermectin and withdrawal of immunosuppressive therapy are the treatments of choice for* Strongyloides *colitis [11]. In contrast, IBD is treated with corticosteroids, which has been proven to be the leading risk factor for the most severe forms of strongyloidiasis [1, 2, 4]. Thus, correct diagnosis of* Strongyloides *colitis is fundamental for the selection of an appropriate treatment. Misdiagnosis of IBD is common due to the similarities in clinical presentation, endoscopy, and histology. Attention to subtle morphologic differences between strongyloidiasis and IBD is critical for minimizing diagnostic error. This neglected condition requires a high index of suspicion, especially in patients infected with HTLV-1 or who are undergoing treatment with corticosteroids or chemotherapy. Initiation of antihelminthic therapy for* Strongyloides *colitis and withholding immunosuppressive medications are crucial for preventing a fatal outcome in this curable disease.
